# Barriers between community screening for visual problems and treatments in a tertiary center

**DOI:** 10.11606/S1518-8787.2018052000589

**Published:** 2018-11-22

**Authors:** Gabriel de Almeida Ferreira, Marcelo Abrão Rezende, Roberta Lilian Fernandes de Sousa Meneghim, Silvana Artioli Schellini

**Affiliations:** I Universidade Estadual Paulista. Faculdade de Medicina de Botucatu. Departamento de Oftalmologia, Otorrinolaringologia e Cirurgia de Cabeça e Pescoço. São Paulo, SP, Brasil

**Keywords:** Blindness, rehabilitation, Eye Health Services, Triage, Health Services Accessibility, Tertiary Healthcare, Outcome and Process Assessment (Health Care)

## Abstract

**OBJECTIVE:**

To evaluate the effectiveness of mobile ophthalmic unit screenings and to investigate barriers between community care and resolution of the problem at a tertiary center.

**METHODS:**

This prospective study evaluated a convenience sample from 10 municipalities in São Paulo State, Brazil. Patients were assessed in the municipality by a mobile ophthalmic unit and underwent a complete ophthalmic consultation. Patients were referred as warranted to a tertiary hospital.

**RESULTS:**

The mobile ophthalmic unit screened 1,928 individuals and 714 (37%) were referred. The mean age of the referred patients was 57.12 (SD = 19.5) years with best corrected visual acuity of 0.37 (SD = 0.36) logMAR. Forty-seven (6.6%) patients were blind and 185 (26.5%) were visually impaired. Cataracts (44.7%) and pterygium (14.7%) accounted for most referrals. Of those referred, 67.1% presented to the tertiary center. The diagnosis by the mobile ophthalmic unit corresponded to the one by the tertiary center in 88.5% of the cases. There were a significantly higher number of blind and visually impaired persons among those who presented to the hospital. There was a significantly greater attendance among patients living in more distant municipalities from the reference center with a higher number of inhabitants and a greater number of ophthalmologists in the cities of origin (p < 0.05, all comparisons). Complete treatment was performed in 65.6% of patients, and loss to follow-up was the main cause of incomplete treatment in 50.7% of patients. A total of 313 cataract surgeries were performed, which reduced the number of blind patients from 20 to 2 and of visually impaired individuals from 87 to 2 (p < 0.001).

**CONCLUSIONS:**

Only 37% of the patients assessed by a mobile ophthalmic unit required referral to a tertiary hospital. Among the referred patients, 67.1% presented to the hospital, and complete resolution after treatment was approximately 65.5%. There was a significant improvement in visual acuity and a reduction in the prevalence of blindness and visual impairment postoperatively.

## INTRODUCTION

Vision-related conditions are increasing worldwide because of demographic transition and aging populations ^1^ . In 2010, the World Health Organization (WHO) estimated that 32.4 million persons were blind globally (0.5% of the world population) and 191 million were visually impaired (2.8 % of the world population) ^2^ .

A lack of optical correction is responsible for 43% of the visual impairment followed by cataract (33%) ^3^ . Both of them are considered reversible causes of visual impairment. However, barriers to access health services may also be a factor in addressing these causes. These barriers can be related to the patient (unperceived low vision, lack of a companion, and fear of consultation or surgery) or services (lack of financial conditions or lack of accessibility) ^4^ .

The Brazilian Unified Health System (SUS) is a public and universal health system. However, ophthalmologic coverage in Brazil is suboptimal because of the large land mass of the country and socioeconomic and developmental differences. Mobile ophthalmic units (MOU) represent a means of overcoming some barriers, which can facilitate the population access to ophthalmic care in underserved regions. Most eye diseases can be treated with a properly equipped MOU, and the remaining conditions that require specialized or surgical treatment can be referred to specialized hospitals.

The MOU are a relative recent project in Brazil. This study evaluates the effectiveness of the care using MOU screening in the community. Additionally, this study investigated the barriers between community care and the resolution of the ophthalmic problem at the reference center at a tertiary hospital.

## METHODS

This prospective study was approved by the Research Ethics Committee of the *Faculdade de Medicina* of the *Universidade Estadual Paulista* , São Paulo, Brazil, and it adhered to the tenets of the Declaration of Helsinki. All patients who participated were required to sign an informed consent form.

The study had a convenience sample comprised of subjects with ocular complaints from 10 municipalities of the central-western region of the state of São Paulo, Brazil, in 2015 ( Table 1 ). All patients were screened by the MOU in the community, and they received optical prescription or clinical treatment whenever needed. Patients who needed specialized clinical or surgical treatment were referred to the Clinic Hospital of the *Faculdade de Medicina de Botucatu* (CH-FMB), which is considered the reference center for the region where this study was conducted.

**Table 1:** Demographic data of municipalities visited by the mobile ophthalmology unit in 2015.

Municipalities	Screening date	Population	per capita Income (R$)	per capita GDP (R$)	HDI	Distance to hospital (km)	Number of ophthalmologists
Mineiros do Tietê	2/2/2015 and 24/8/2015	12,038	694.22	10795.16	0.730	67.1	0
Piramboia	9/2/2015	5,653	549.16	17558.27	0.721	44.2	0
Taquarituba	25/5/2015	22,291	613.84	19516.97	0.701	139	1
Igaraçu do Tietê	1/6/2015	23,362	587.44	10229.66	0.727	50.6	0
Dois Córregos	15/6/2015	24,761	720.28	20050.38	0.725	76.8	2
Boracéia	29/6/2015	4,268	708.05	30521.77	0.754	102	0
Bariri	20/7/2015	31,593	771.49	24914.02	0.750	110	2
Macatuba	27/7/2015	16,259	918.61	26688.61	0.770	65.3	0
Brotas	3/8/2015	21,580	711.01	22964.88	0.740	98.4	0
Barra Bonita	9/11/2015	35,246	903.18	16523.66	0.788	53.8	4

GDP: gross domestic product; HDI: human development index.

All care was free of cost, provided by the SUS. The MOU was comprised of two ophthalmologists, two ophthalmic technicians, and three ophthalmology residents. The MOU evaluated an average of 150 patients per visit in each municipality. The transportation to the CH-FMB was provided by the municipalities of origin without costs.

Data were collected on demographics of the municipality of origin [per capita GDP (gross domestic product), average per capita family income, and number of inhabitants, provided by the Brazilian Institute of Geography and Statistics ^5^ ^,^ ^6^ ], human development index (HDI) based on the United Nations Development Programme definition ^7^ , and number of ophthalmologists according to the SUS Department of Informatics ^8^ . Data were also collected on the ophthalmic care performed at the reference center including the time between MOU screening and patient presentation to the reference center, the number of consultations performed, treatment proposed and performed, number of surgeries, complications, and outcomes. Outcomes were estimated by corrected visual acuity (VA) or resolution of the chief complaint.

Patients were included if they were evaluated in 2015 by the MOU in municipalities of the central-western region of São Paulo State and if they were suspected of having ophthalmic diseases and needed referral to the tertiary center. Exclusion criteria were refusal to participate in the study and lack of information in the electronic medical records.

The MOU was equipped with two manual refractors (RT 6000; NIDEK Co. Ltd., Gamagori, Japan), two optotype projectors (ES-03 Xenônio, São Paulo, Brazil), two slit lamps (Shinn Nippon Corp., Tokyo, Japan), two Goldmann applanation tonometers (Haag-Streit Holding, Köniz, Switzerland), one non-contact tonometer (CT-60; Topcon Corp., Tokyo, Japan), one autorefractor (Accuref - K Shinn Nippon Corp., Tokyo , Japan), two 78 D lenses (Volk Optical Inc., Mentor, Ohio, USA), two retinoscopes (Welch Allyn Inc., Skaneateles Falls, NY, USA), and three Snellen eye charts. The patients underwent an ophthalmic examination consisting of VA measurement with and without optical correction using the Snellen chart at a distance of five meters. If the patient could not see the largest symbol in the Snellen chart, VA was checked with finger counting, hand movement, and light perception. The VA was converted to logMAR for statistical analysis with the values of 2.10, 2.40, 2.70, and 3.00 logMAR corresponding to finger count, hand movement, light perception, and no light perception, respectively ^9^ . A torch light was used for external examination. Absence or presence of strabismus was checked with the Hirschberg test and the simple cover and alternating cover tests. Slit lamp examination was performed on the anterior segment and posterior segment; biomicroscopy was performed with a 78 D fundus lens (under medication-induced mydriasis, when necessary). Intraocular pressure was measured with air-puff tonometry followed by Goldmann applanation tonometer if the air-puff tonometer detected intraocular pressure exceeding 20 mmHg ^10^ .

Examination at the reference center was the same as at the MOU. However, additional diagnostic tests were performed including biometry (IOLMaster 500; Carl Zeiss Meditec, Jena, Germany). All cataract surgery cases were targeted for emmetropia. If dense cataracts precluded optical biometry, axial length was measured with an ultrasonic contact biometer (SP-1000AP; Sonoptek, China) and the intraocular lens (IOL) power was calculated on the IOLMaster 500.

Blindness and visual impairment were defined according to WHO criteria, with blindness classified as VA < 20/400 and visual impairment as 20/400 < VA < 20/60 in the best eye and with the best optical correction ^11^ .

We defined a fully completed treatment when patients underwent the entire proposed treatment, both surgical and clinical. In the surgical cases, bilateral treatment was considered as indicated. If patients were awaiting evaluation or a surgery at the reference center, the treatment was classified as partial.

All data were tabulated in a spreadsheet and transferred to SPSS 22.0 software (IBM Corp., Armonk, NY, USA) for analysis. The results were evaluated according to frequency of occurrence, mean, and standard deviation. The normal distribution was analyzed by the Kolmogorov-Smirnov and Shapiro-Wilk tests and the subsequent analysis was performed with the relevant tests. The continuous variables are expressed by mean and standard deviation. Statistical significance was classified as p < 0.05.

## RESULTS

**Figure 1:**
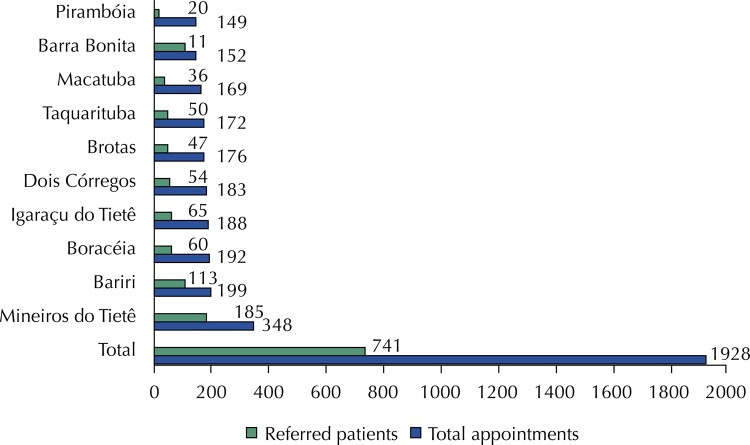
Patients referred by a mobile ophthalmic unit for treatment at a tertiary center in 2015.

This study enrolled 1,951 individuals, of whom 23 were excluded because of a lack of information in the medical records. Thus, 1,928 individuals were evaluated, of whom 714 (37%) required referral to the tertiary center, which ranged from 13.4% to 73% according to the municipality of origin ( Figure 1 ). The mean age of the referred patients was 57.1 (SD = 19.5) years (median = 62 years, ranging from one to 90 years) and 428 (59.9%) were females.

The VA was determined in 699 (97.9%) patients. Mean uncorrected VA (UCVA) in the best eye was 0.54 (SD = 0.56) logMAR (approximately 20/60) and mean best corrected VA (BCVA) in the best eye was 0.37 (SD = 0.36) logMAR (approximately 20/50). Forty-seven (6.6%) patients were blind and 185 (26.5%) were visually impaired. There was a statistically significant association between VA and age, in which VA was worse in older individuals (r = 0.38, p < 0.001). Blind and visually impaired persons were older, 66.2 (SD = 18.9) and 66.5 (SD = 14.4) years respectively, compared to patients with normal VA [53.5 (SD = 18.6) years, p < 0.001].

Three hundred and nineteen (44.7%) patients were referred for cataract, followed by 107 (15%) patients for pterygium and 49 (6.9%) patients for suspicion of glaucoma ( Table 2 ).

**Table 2:** Ophthalmic disorders and referral of patients by a mobile ophthalmic unit to a tertiary center in 2015.

Disorders	n	%
Crystalline disorders	349	48.9
Eyelid disorders	196	27.4
Glaucoma	61	8.5
Retinal disorders	41	5.7
Corneal and ocular surface disorders	27	3.8
Strabismus	20	2.8
Refractive changes	8	1.1
Orbital disorders	7	1.0
Uveal disorders	5	0.7

Of the 714 referred patients, 479 (67.1%) presented to the tertiary (reference) center and eight (1.1%) were still waiting consultation at the time of data collection and were not included in the analysis.

Among the patients who presented, the main reason for referral to the reference center was surgical treatment (349 patients; 72.9%), followed by clinical follow-up (n = 83; 17.3%), laser treatment for posterior capsule opacification or retina (n = 46; 9.6%), or botulinum toxin treatment (n = 1; 0.2%).

The diagnosis from the MOU and tertiary center was the same in 88.5% (424 of 479) of the cases. Most differing diagnoses corresponded to the indication of cataract surgery, in which, after detailed examination and complementary tests, another ocular comorbidity was detected, which contraindicated the surgical treatment (21 cases; 38.2% of the differing diagnoses).

**Figure 2:**
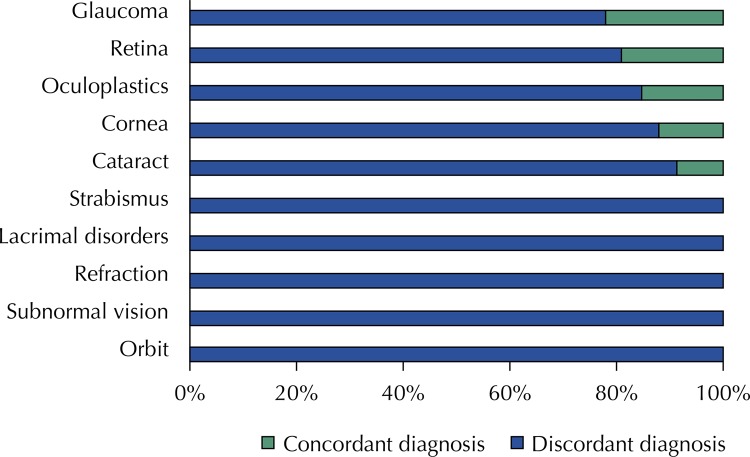
Distribution of the agreement in diagnoses between a mobile ophthalmic unit and a tertiary center according to subspecialty in 2015.

For the ophthalmic specialties, the lowest agreement between the diagnoses from the MOU and reference center were glaucoma (78.3%) and retina (81.1%) ( Figure 2 ).

Age, sex, and BCVA in the best eye were not determinants for presentation to the tertiary service (p > 0.005). There was a statistically significantly higher frequency of blindness (7.5% versus 5.4%) or visual impairment (29.2% versus 21.6%) among patients who presented to the reference center versus those who failed to present (p = 0.035).

According to the demographics of the municipalities of origin, there was a statistically significantly greater attendance among patients living in more distant municipalities from the reference center (81.9 km versus 77.3 km, p = 0.034), with a higher number of inhabitants (22,449.2 versus 19,477.3 inhabitants, p < 0.001), and with a greater number of ophthalmologists in the cities of origin (1.30 versus 0.87 ophthalmologists, p < 0.001). The HDI, per capita GDP, and average per capita family income did not influence who presented to the reference center.

The mean time interval between presentation to the MOU and consultation at the reference center was 133.2 (SD = 113.1) days. The mean time between the consultation and the necessary surgical procedure was 55.5 (SD = 55.4) days.

After examination at the reference center, 3.8% of the patients did not require any further clinical follow-up or surgery. Among those who were referred, 65.6% underwent complete treatment, 20% had partial treatment, and 10.4% received no treatment.

The main causes of partial or non-treatment were loss to follow-up (50.7%), waiting for the procedure (36.3%), the patient refused the procedure (9.6%), and only clinical follow-up was indicated (3.4%).

A total of 204 (58.8%) individuals underwent cataract surgery (313 total cataract surgeries), with an average of 5.3 (SD = 3.1) visits per patient. The mean time between referral by the MOU and care in the cataract clinic was 81.2 (SD = 51.2) days. The mean time between the consultation and the cataract procedure was 57.8 (SD = 60.9) days with an average follow-up of 166.8 (SD = 103) days.

Complications occurred in 46 (14.7%) cataract surgeries. Posterior capsule rupture was the most common complication accounting for 39.1% of them (5.7% of the surgeries) ( Table 3 ).

**Table 3:** Complications of cataract surgeries performed at a tertiary reference center in patients referred by a mobile ophthalmic unit in 2015.

Complications	n (% of complications)	% of total surgeries
Posterior capsular rupture	14 (30.4)	4.5
Postoperative positive Seidel	6 (13)	1.9
Corneal decompensation or bullous keratopathy	5 (10.9)	1.6
Zonular disinsertion	5 (10.9)	1.6
Corneal stitch	4 (8.7)	1.3
Posterior dislocation of nucleus	4 (8.7)	1.3
Descemet detachment	2 (4.3)	0.6
Capture of intraocular lens	1 (2.2)	0.3
Iridoplasty	1 (2.2)	0.3
Expulsive hemorrhage	1 (2.2)	0.3
Postoperative cortical rest	1 (2.2)	0.3
Cystoid macular edema	1 (2.2)	0.3
Iris herniation	1 (2.2)	0.3

Data on final VA was available in 149 (73%) cataract patients who underwent surgery. The BCVA improved statistically significantly from 0.63 (SD = 0.57) logMAR at presentation to 0.11 (SD = 0.29) logMAR at the last visit (p < 0.001). Initially, 20 (10%) patients were considered blind and 87 (43.5%) were visually impaired. After surgery, there was a statistically significant improvement and only two blind (1%) and two (1%) visually impaired patients remained (p < 0.001).

## DISCUSSION

The outcomes of this study indicate that, on average, 37% of the patients among all evaluated municipalities were referred to the specialized ophthalmic service after MOU screening. However, the referral rate greatly varied among municipalities. This variation was likely due to the different profile of the municipalities and different levels of demand.

The evaluation of referrals to specialized ophthalmology services after ophthalmic screening is a subject rarely reported in the literature. A single Brazilian study reported that 8.9% of 1,010 patients were referred to a primary unit in six months ^12^ . However, in this study, there was no pent-up demand for services. A Canadian study has reported that, in three years, 9% of routine eye visits required referral to a specialized unit ^13^ . These percentages are much lower than the ones in our study, which evaluated municipalities with poor access to ophthalmic services and those with a previous pent-up demand for services.

In this study, mean BCVA in the best eye was 0.37 (SD = 0.36) logMAR (approximately 20/50). A British study has reported that the preoperative VA of cataract patients was 0.63 logMAR ^9^ . However, that study addressed only patients with cataract, which evidently reduces mean VA. Additionally, in our study, VA was worse in older patients, in addition to a higher burden of blindness and visual impairment, which can be explained by the higher prevalence of cataract, age-related macular degeneration, and other diseases that decrease VA in this age group.

Our study evaluated a convenience sample comprised of individuals of all ages with ocular complaints. At the MOU screening, 47 (6.6%) individuals were blind and 185 (26.5%) were visually impaired. Studies enrolling randomized samples have reported a 2.14% prevalence of visual impairment in patients over 40 years of age and a 0.68% prevalence of blindness in the United States of America ^1^ . However, others have reported greater prevalence of blindness in sub-Saharan Africa (5.7%), North Africa (4.6%), Middle East (4.6%), and South Asia (4.4%), which are likely due to inequality in health care in different regions of the world ^2^ .

A study conducted in São Paulo, SP, Brazil, has reported a prevalence of blindness of 1.1% and a prevalence of visual impairment of 2% ^14^ . A Pan-American study using WHO criteria has evaluated patients over 50 years of age in some countries and it has reported that the average prevalence of blindness ranged from 0.7% in Argentina to 3% in Panama, and visual impairment ranged from 8% to 14.3% in Uruguay and El Salvador, respectively ^15^ .

Cataract remains a major cause of global blindness ^11^ ^,^ ^16^ ^,^ ^17^ . In our study, the most common indication for referral to the tertiary hospital was cataract (44.7% of referrals). The prevalence of cataracts in Europe increases with age, which ranges from 5% for individuals aged 52 to 62 years, 30% for individuals aged 60 to 69 years, to 64% for individuals over 70 of age ^18^ . In a Chinese population with a mean age of 52 (SD = 11.8) years, the prevalence of cataract was 20.8% ^19^ .

In our study, the second most common condition warranting referral was pterygium (15% of the referrals). Pterygium often requires surgical treatment and advanced cases may cause decreased VA.

A study similar to ours by US Army physicians also with a convenience sample of individuals has shown that most cases (45.3%) warranting referral were due to anterior segment disorders, followed by oculoplastic problems (23.9%) ^20^ . Hence, despite the different profile of the patients, the distribution of the pathology was similar to our study.

In our study, 67.1% of the patients referred by the MOU were presented to the reference center. Most of these patients were referred for a surgical procedure (72.9%). This percentage of presentation to the tertiary center was considered low, as several actions were taken to facilitate it, such as the care was performed in the municipality of origin, the referral was directed to the specialty hospital, and transportation was provided by the health municipalities. The rate of patient presentation to the referral hospital or center after screening has not been reported in the literature. Distance to the referral center, fear of surgery, transportation and expenses, lack of companionship, and the expense of surgery have been reported as barriers to cataract surgery ^21^ ^,^ ^22^ . However, some of these reasons are not valid for our study population as the SUS covers the cost of surgery and transportation is guaranteed by the municipalities.

In this study, the factors that influenced non-attendance to the referral center were the absence of blindness or visual impairment, shorter distance to the tertiary service, lower number of inhabitants, and fewer ophthalmologists in the municipalities of origin. The greater attendance rate of blind and visually impaired patients seems obvious because of the personal burden of the reduced vision. The lower attendance of patients from the nearest municipalities may be because they can easily access the service outside of the screening campaigns and small municipalities theoretically have a smaller structure, which would hinder the presence of free transportation.

The diagnoses from the MOU and the reference center were the same in 88.5% of the cases, which reinforces the reliability of the ophthalmic examination performed by the MOU and the possibility of diagnosing several diseases using basic ophthalmic equipment and a rapid examination.

Of the diverging diagnoses between the MOU and the reference center, most (21/55 or 38.2%) were due to cataract cases in which surgery was contraindicated because of the identification of concomitant diseases after complementary examinations in the reference center.

The lowest agreement in diagnosis between the MOU and the reference center was for glaucoma (78.3%) and retinal disease (81.1%). In the former case, glaucoma suspects were also referred, hence complementary tests are needed to include/exclude glaucoma suspects. Additionally, glaucoma is a diagnosis that depends on physician experience^23–26^. A study ^27^ of telemedicine-based referrals has reported greater disagreement in glaucoma and retinal diagnoses, which is similar to our study.

Treatment at the CH-FMB (the reference center) was considered as completed in 65.6% of the cases, which seems unsatisfactory. A total of 34.4% had only partial treatment; however, 50.7% of them were lost to follow-up. Therefore, if there was no loss to follow-up, the treatment rate could have approached 80%, which is considered adequate.

This outcome indicates that patients seem to have difficulty going for their initial consultation and maintaining regular follow-up. The same factors that influence the initial consultation visit may also influence adherence to other consultations. Some of these factors are distance to the reference center, lack of a companion to take the individual to the consultation, and lack of transportation.

A total of 313 cataract surgeries were performed at the reference center and complications occurred in 14.7% of the cases. The main complication was posterior capsule rupture, which occurred in 5.7% of the surgeries. Previous studies have reported posterior capsule rupture rates from 1.8% to 3.06% ^28^ ^,^ ^29^ . A much higher rate has been reported in another study of ophthalmologists in training (29%) ^30^ . The rate of posterior capsule rupture in our study can be considered satisfactory because CH-FMB is a teaching hospital with procedures being performed by fellows in training and because it is a reference center with a higher frequency of complicated cases.

In this study, there was a statistically significant reduction of visual impairment and blindness after cataract surgery. For example, there was a reduction of visual impairment from 43.5% at the initial screening to 1% postoperatively. Similarly, there was a reduction of blindness from 10% at the initial screening to 1% postoperatively.

Our findings also indicate that the MOU can reliably detect the most frequent ophthalmic conditions using simple examination techniques and low financial resources, which reinforces the concept that MOUs are very useful for ophthalmic screening within the SUS.

An important limiting factor of our study was the lack of integration between local ophthalmologists and the team that visited the municipality, which can also represent a barrier to non-attendance and loss to follow-up. Local doctors have direct interaction with patients and their participation would facilitate the referral back to the municipality and improve the chances of follow-up.

In conclusion, the attendance rate of patients referred directly from a MOU to a tertiary center was 67.1%. The main causes for referral were cataract and pterygium. The agreement between the diagnoses by the MOU and the tertiary service was 88.5%. In addition, 65.5% of the cases underwent complete treatment. After cataract surgery, the burden of visual impairment and blindness decreased appreciably.
